# The role of mitotype variation and positive epistasis in trait differences between *Saccharomyces* species

**DOI:** 10.1093/genetics/iyaf233

**Published:** 2025-10-27

**Authors:** Jun-Ting Johnson Wang, Ping Ling Priscilla Ng, Maceo E Powers, Catherine H Rha, Rachel B Brem

**Affiliations:** Department of Plant and Microbial Biology, University of California, Berkeley, Berkeley, CA 94720, United States; Graduate Group in Comparative Biochemistry, University of California, Berkeley, Berkeley, CA 94720, United States; Department of Plant and Microbial Biology, University of California, Berkeley, Berkeley, CA 94720, United States; Department of Plant and Microbial Biology, University of California, Berkeley, Berkeley, CA 94720, United States; Department of Plant and Microbial Biology, University of California, Berkeley, Berkeley, CA 94720, United States; Department of Plant and Microbial Biology, University of California, Berkeley, Berkeley, CA 94720, United States

**Keywords:** trait variation, evolution, mitochondrial divergence, positive epistasis

## Abstract

Many traits of interest in biology evolved long ago and are fixed in a particular species, distinguishing it from other sister taxa. Elucidating the mechanisms underlying such divergences across reproductive barriers has been a key challenge for evolutionary biologists. The yeast *Saccharomyces cerevisiae* is unique among its relatives for its ability to thrive at high temperature. The genetic determinants of the trait remain incompletely understood, and we sought to understand the role in its architecture of species variation in mitochondrial DNA. We used mitochondrial transgenesis to show that *S. cerevisiae* mitotypes were sufficient for a partial boost to thermotolerance and respiration in the *Saccharomyces paradoxus* background. These mitochondrial alleles worked best when the background also harbored a pro-thermotolerance nuclear genotype, attesting to positive epistasis between the two genomes. The benefits of *S. cerevisiae* alleles in terms of respiration and growth at high temperature came at the cost of worse performance in cooler conditions. Together, our results establish this system as a case in which mitoalleles drive fitness benefits in a manner compatible with, and fostered by, the nuclear genome.

## Introduction

A central aim in evolutionary genetics is to figure out how organisms acquire new traits. In the study of within-species variation, a rich literature has provided insights into the mechanisms of local adaptation in the wild (Elena 2017; Kraemer and Boynton 2017; Sork 2017; Rees et al. 2020; Bazzicalupo 2022). Yet many characters of basic and applied interest evolved long ago and are fixed in a particular species, distinguishing it from unaffected relatives. Against a backdrop of years of genomic and experimental pursuit (Allen Orr 2001; Nikolov and Tsiantis 2015; Weiss and Brem 2019), the genetic principles governing trait divergence over long timescales remain poorly understood, with the widest knowledge gap in the case of polygenic architectures. Indeed, an adaptive trait that arose long ago, and ultimately fixed in a given species, could have undergone millions of years of refinement, and could have a genetic architecture governed by principles quite different from the recently arisen intra-species trait polymorphism that is the focus of much of the field. Shedding light on these mechanisms, by dissecting the genetics of adaptations between isolated taxa, continues to pose challenges for the field.

Sequence divergence in mitotype, the DNA of the mitochondrial organelle, have emerged as an important driver of naturally varying traits. Mitotype variants have been directly implicated in trait diversity, both independently and in concert with nuclear genomes, in a rich literature focused on polymorphism within species (Melvin and Ballard 2006; Tranah 2011; Houtkooper et al. 2013; Sullivan and Chandel 2014; [Bibr iyaf233-B70][Bibr iyaf233-B72]; Patel et al. 2016; Camus et al. 2017; Camus and Dowling 2018; Mossman et al. 2019; Sun et al. 2019; Camus et al. 2020; Salminen and Vale 2020; Anderson et al. 2022; Cao et al. 2022; Meng et al. 2022; Oppong et al. 2022; Quéméneur et al. 2022; Parra et al. 2023; Moran et al. 2024). Though small in length, the mitochondrial genome has the potential for an outsize influence on trait variation, given the increased mutation rate and large mutational target of the multicopy mitochondrial genome relative to nuclear chromosomes (Pakendorf and Stoneking 2005; Chou and Leu 2015; Ferreira and Rodriguez 2024).

*Saccharomyces* budding yeasts diverged from a common ancestor ∼20 million years ago (Kellis et al. 2003) and have served for decades as a model for the study of interspecific trait variation, including in metabolism, genome content, cell cycle, and reproductive isolation (Hou et al. 2014; Roop et al. 2016; Bozdag et al. 2021; Fredericks et al. 2021; Lupo et al. 2021; Marsit et al. 2021; Smukowski Heil et al. 2021; Swamy et al. 2022; Crandall et al. 2023; Forejt and Jansa 2023; Peris et al. 2023). A long-standing subset of this field has focused on species-unique traits driven by mitochondrial variation (Sulo et al. 2003; Wolff et al. 2014; De Chiara et al. 2020; Hewitt et al. 2020; Hénault et al. 2022), with particularly detailed mechanistic validation in studies of speciation (Lee et al. 2008; Chou et al. 2010; Mário et al. 2015; Jhuang et al. 2017) (complementing studies of mitotype polymorphism within yeast species [Wolters et al. 2015; Leducq et al. 2017; Wolters et al. 2018; Vijayraghavan et al. 2019; Vijayraghavan et al. 2019; Nguyen et al. 2020]). A key model trait in the *Saccharomyces* system is temperature preference, as the best-studied yeast species, *S*. *cerevisiae,* is unique within the *Saccharomyces* clade for its ability to thrive at high temperature (Sweeney et al. 2004; Gonçalves et al. 2011; Salvadó et al. 2011), whereas others in the clade exhibit cryotolerance (Paget et al. 2014; Pinto et al. 2025). Species-level variation in these traits has been inaccessible to analysis by classic methods like association or linkage mapping, which rely on recombinants in fertile crosses. For this reason, mechanisms by which nature built thermotolerance in *S. cerevisiae* from a thermosensitive ancestor have been difficult to access for the classic literature. But the system represents a testbed for alternative approaches for genetic dissection across species boundaries, which have enabled insight into the architectures of species differences in thermotolerance and their adaptive history (Weiss et al. 2018; AlZaben et al. 2021; Walunjkar et al. 2025) and cold tolerance (Paget et al. 2014; Pinto et al. 2025). The contribution of mitochondrial genome variation to this trait has also been suggested, based on analyses of interspecies hybrids (Baker et al. 2019; Li et al. 2019; Hewitt et al. 2020); exactly what the divergent mitotypes of *Saccharomyces* species do phenotypically, and how they exert their effects, remains incompletely understood.

We set out to pursue in more depth the phenotypic impact of mitochondrial variation between *Saccharomyces* species, both in isolation and in conjunction with nuclear loci. We anticipated that our findings would further illuminate the mechanisms of thermotolerance and their evolutionary history. We designed an approach that used *S. paradoxus*, a relatively thermosensitive species sister to *S. cerevisiae*, as a foreign background for manipulations of the nuclear and mitochondrial genomes, to investigate their functions and their interactions.

## Materials and methods

### Strain construction

Strains used in this study are listed in Supplementary Table 1. For phenotyping in Supplementary Fig. 1, we used wild-type, homozygous diploid *S. cerevisiae* strains DBVPG1373, YPS128, DBVPG6044 and Y55, and *S. paradoxus* strains Z1, UFRJ50816, A4 and KPN3828, from the Saccharomyces Genome Resequencing Project (SGRP) collection (Supplementary Table 1; Cubillos et al. 2009). To make haploid strains for the recipients for *S. cerevisiae* mitochondrial DNA, we first generated prototrophic versions of diploid *S. paradoxus* Z1 and its transgenic descendant, a Z1 derivative harboring the eight *S. cerevisiae* loci, designated as 8X (AlZaben et al. 2021). For this, the *HO* loci of the diploid wild-type Z1 and 8X strains were knocked out using the hygromycin resistance gene as the selection marker using the transformation protocol from (Reuß et al. 2004), with selection on 200 μg/mL hygromycin and PCR and sequence confirmation. The resulting *hoΔ*::Hyg diploid strains were induced to sporulate essentially as in (Lee et al. 2008), and mating types of the resulting haploids were checked via halo assay following the protocol in (Hoffman et al. 2002), using JRY02375 and JRY02176 (a generous gift from Dr. Jasper Rine) as the mating testers. Next, for haploid MATα *S. paradoxus* Z1 and its descendant 8X, we generated a petite version of each (lacking mitochondrial DNA, ρ^0^), using the protocol adopted from Amine et al. (2021). In short, yeast cells were streaked from −80 °C stocks on yeast peptone dextrose (YPD) plates and grown at 28 °C for 2 days. A colony was inoculated into 3 mL of YPD and incubated at 28 °C overnight. 10^4^ cells were inoculated into 1 mL of YPD supplemented with ethidium bromide (10 μg/mL) and incubated at 28 °C overnight. Cells were harvested and washed twice with ddH_2_O. About 200 cells were plated onto YPD plates and grown at 28 °C for 2 to 3 days to form visible colonies. The plates were replicated to yeast peptone glycerol (YPG) plates to look for colonies that lost the ability to respire, ie those in the ρ^0^ state.

Finally, we used wild-type, MATα haploid, G418-resistant, hygromycin-resistant *S. cerevisiae* strains DBVPG1373, YPS128, DBVPG6044, Y55 and DBVPG6765 from the SGRP collection (Cubillos et al. 2009), and MATα haploid hygromycin-resistant *S. cerevisiae* strains (SJ6L01, Sx3 and GE14S01-7B), a generous gift from Joseph Schacherer (Supplementary Table 1; Peter et al. 2018), as donors of mitochondrial DNA for cybrids in the *S. paradoxus* wild-type (Z1), 8X (Z1 with 8 *S. cerevisiae* nuclear loci), and *S. cerevisiae* (DBVPG1373) backgrounds. Our method for cybrid construction used the cytoduction protocol of Nguyen et al. (2020) as follows. We first constructed a heterokaryon-competent *S. cerevisiae* strain harboring each mitotype of interest, referred to as a carrier. For this, a single colony of each donor in turn, and a single colony of the ρ^0^  *kar1*-d15 mutant (JRY5450, a generous gift from Dr. Jasper Rine), were each inoculated into a separate 3 mL of liquid YPD (1% yeast extract, 2% peptone, 2% glucose) and incubated at 28 °C overnight with shaking at 250 rpm. We mixed 100 μL of the two, spotted on a YPD plate, and incubated at 28 °C for 4 h. The mixed cellular patch was inoculated into 3 mL of YPD and incubated at 28 °C for 2 h with shaking at 250 rpm to promote cell division. After a 2-h incubation, 10^−5^ OD_600_ of cells were spread on a YPG plate (1% yeast extract, 2% peptone, 2% glycerol) to select for mitochondrial genome retention. Colonies on the YPG plates were transferred to a YPD plate with 200 μg/mL hygromycin or 200 μg/mL G418 to check for hygromycin and G418 sensitivity; we earmarked and preserved drug-sensitive, respiration-competent strains as those in which the donor mitochondrial DNA had successfully been transferred to the JRY5450 nuclear background. We next used each carrier strain in turn to transfer the *S. cerevisiae* mitochondrial DNA of interest into the haploid *S. paradoxus* Z1 ρ^0^ strain and, separately, into its descendant, the 8X ρ^0^ strain. This cytoduction proceeded as above except that the carrier and the *S. paradoxus* strain were mated and the progeny were selected on YPG alone; afterward we subjected them to the halo assay, to eliminate the carrier-*S. paradoxus* diploid hybrids, and only those strains that behaved as haploid MATα were retained as cybrids.

### Growth assays

Measurements of cell density were done as described (AlZaben et al. 2021) with modifications as follows. To make sure the cells were respiratory-competent before the assay, strains were streaked on YPG plates and were allowed to grow at 28 °C for 4 days to form colonies. Three colonies were inoculated into 15 mL of liquid YPG and incubated at 28 °C for overnight with shaking at 250 rpm. The cultures were then back-diluted into 5 mL of YPD, YPE (1% yeast extract, 2% peptone, 2% ethanol), or YPG to achieve 0.1 OD_600_/mL, and then incubated at 23, 28, or 39 °C for 24 h. This procedure, from streaking on solid agar plates to biomass measurement, was repeated at least twice for each strain with three technical repeats in each biological replicate. We collected the readouts of cell density from all the replicates across all days for a given strain and compared each mitochondrial transgenic to *S. paradoxus* with a two-tailed Mann–Whitney U test. For epistasis analysis, a two-way ANOVA test was carried out to examine the impact of the genotype–mitotype interaction on thermotolerance. All statistical analyses were done with scipy.stats in Python 3.7. Data and statistical analyses for all growth assays in this study are reported in Supplementary Table 2.

### MTT assay

To measure respiration efficiency, preculture, and treatment were as in Growth assays, above, and after 24 h we carried out the MTT assay as in (Sánchez and Königsberg 2006). Independent experiments from growth to MTT measurement were performed at least six times. The readouts were normalized to that of wild-type *S. paradoxus* at either 28 or 39 °C. Two-tailed one-sample Wilcoxon test was performed using the normalized data with a hypothesized value of 1 with scipy.stats in Python 3.7. Data and statistical analyses for MTT assays are reported in Supplementary Table 2.

### Growth of *S. cerevisiae* and *S. paradoxus* in glycerol

Data are from Warringer et al. (2011). The Mann–Whitney U test was performed to compare the growths of the two species in glycerol with scipy.stats in Python 3.7. Raw data and statistical analyses are reported in Supplementary Table 2.

### Trehalose and glycogen assays

To measure the contents of trehalose and glycogen stored in yeast, we took advantage of the protocol from Chen and Futcher (2017). Briefly, yeast cells were pregrown in YPG overnight. 0.3 OD_600_ of cells were harvested, and the pellets were washed with 1 mL of ddH_2_O to remove residual media. The pellets were resuspended in 125 μL of 0.25 M Na_2_CO_3_ solution, and incubated at 95 °C for 3 h in PCR tubes. Seventy-five microliters of 1 M acetic acid and 300 μL of 0.2 M sodium acetate, pH 5.2 were added to the mix. The mix was immediately divided into two 250-μL aliquots. For glycogen measurements, 10 μL of *Aspergillus niger* α-amyloglucosidase (20 mg/mL in 0.2 M sodium acetate, pH 5.2) was added to one of the aliquots, and the mix was incubated at 57 °C overnight. For trehalose measurements, 15 μL of 0.2 M sodium acetate, pH 8 was added to the other aliquot to adjust the pH to ∼5.8, and then 3 μL of porcine trehalase was added to the sample, followed by incubation at 37 °C overnight. After incubation, the content of glucose, digested from either trehalose or glycogen, in each sample was measured using the Glucose (GO) Assay Kit (Cat. No. GAGO20) at absorbance of 540 nm. OD_540_ readouts in this setting represent the content of glucose in each sample, reporting the concentration of the substrates, trehalose, and glycogen, in the cells. Data and statistical analyses for trehalose and glycogen assays are reported in Supplementary Table 2.

### Multiple sequence alignment

*S. cerevisiae* mitochondrial genomes were from De Chiara et al. (2020). North American *S. paradoxus* mitochondrial genomes (NCBI accessions: KY287641-KY287662) were from Leducq et al. (2017), and European and Asian *S. paradoxus* genomes (NCBI accessions: JQ862335.1, KP712799.1, KP712802.1, KP712786.1, KP712796.1) were from Procházka et al. (2012). Multiple sequence alignment was performed using Muscle v. 5.1. (Edgar 2022), the allele frequency was calculated and plotted with Python 3.7, and the alignment was visualized with AliView v. 3.0 (Larsson 2014).

## Results

### *S. cerevisiae* mitotypes are sufficient to heighten respiratory behaviors in *S. paradoxus*

**Fig. 1.**
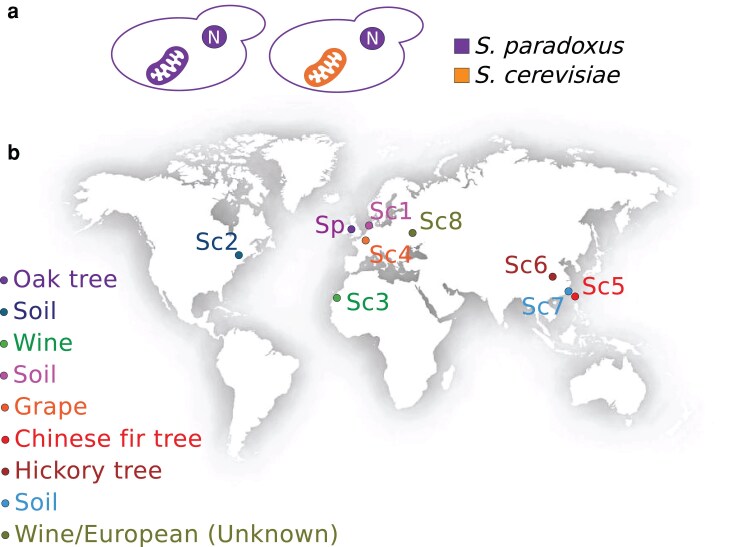
Experimental design and isolates for genetic dissection of *Saccharomyces* species variation in thermotolerance. a) Wild-type *S. paradoxus* (left) is used as the background for a cybrid (right) harboring the endogenous nuclear genome (N) and the mitochondrial genome from a *S. cerevisiae* donor (orange). b) Strains used in this work: Sp, *S. paradoxus* Z1; Sc1 to Sc8, *S. cerevisiae* DBVPG1373, YPS128, DBVPG6044, Y55, SJ6L01, SX3, GE14S01-7B, and DBVPG6765, respectively.

Previous studies have used yeast interspecies hybrids to establish an association between *S. cerevisiae* mitochondrial alleles and thermotolerance (Baker et al. 2019; Li et al. 2019; Hewitt et al. 2020), which parallels the advantage of *S. cerevisiae* purebreds at high temperature, relative to other species in the clade. We reasoned that investigating mitotype genetics in purebred *S. paradoxus* could help shed additional light on the mechanisms of evolutionary innovation along the *S. cerevisiae* lineage. For this purpose, we developed a series of cybrids, in which we introduced each mitochondrial genome in turn from a suite of *S. cerevisiae* isolates from different geographical locations, which all grew well in standard conditions and at high temperature (Supplementary Fig. 1), into a tester strain of *S. paradoxus*, the English oak tree isolate Z1 (Fig. 1). Since they lack the endogenous *S. paradoxus* mitotype, these strains represented an experimental resource for the study of the phenotypic impact of *S. cerevisiae* mitochondrial alleles in an isogenic background, and they were viable and stable as expected (Sulo et al. 2003; Mário et al. 2015).

**Fig. 2.**
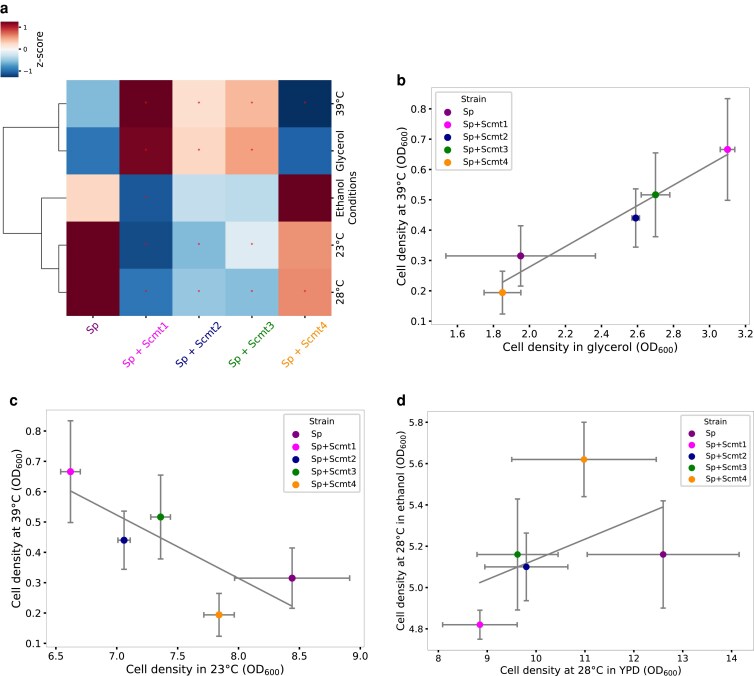
Condition-dependent growth and tradeoffs across cybrids. a) Each element of the heat map reports cell density, as a mean across replicates (*n* ≥ 3), after 24 h of growth in liquid culture in the respective condition, with glucose as the carbon source unless otherwise indicated. Each column reports results from wild-type *S. paradoxus* (Sp) or an isogenic cybrid harboring the mitochondrial genome from a *S. cerevisiae* donor (Scmt) labeled as in Fig. 1. Colors report row-normalized *z*-score. b to d) Shown are re-analyses of cell density measurements across strains from the cultures and conditions in (a), with respective Spearman coefficients of −0.8, 1, and 0.67. In a given panel, each dot reports the median across replicates (*n* ≥ 3), and error bars report standard deviation. Strain labels are as in (a). *, Growth different from that of *S. paradoxus* at two-tailed Mann-Whitney *P* < 0.05. Raw growth measurements, Spearman correlation coefficients, and statistical analyses are reported in Supplementary Table 2a to g.

Before using our cybrids to address thermotolerance per se, we first investigated the hypothesis that respiration, as a proximal function of the mitochondrion and its genome, would be perturbed by species variation in mitotype. Wild-type *S. cerevisiae* strains as a rule grow better than those of *S. paradoxus* on glycerol, a nonfermentable carbon source (Supplementary Fig. 2), and in interspecies hybrid backgrounds, *S. cerevisiae* mitotypes promote growth in this condition (Li et al. 2019). To explore the impact of *S. cerevisiae* mitochondrial DNA alleles on respiratory growth in a purebred context, we assayed growth of our cybrids in liquid glycerol medium. The results revealed robust improvement attributable to *S. cerevisiae* mitotypes in the *S. paradoxus* background, for three of our *S. cerevisiae* donors (Fig. 2a and Supplementary Fig. 3). The fourth cybrid, harboring mitochondrial DNA from the *S. cerevisiae* vineyard isolate Y55, had a defect in growth on glycerol (Fig. 2a), which echoes previously reported incompatibilities of this mitotype in other backgrounds (Wolters et al. 2018). In precultures that served as the inocula for these growth experiments, assays of glycogen and trehalose detected no difference between genotypes, arguing against a role for energy stored by cells from the preculture as a driver of their growth effects (Supplementary Fig. 4). Together, these data suggested that pro-respiratory effects could be a prevalent characteristic of mitochondrial genomes from across the *S. cerevisiae* population.

**Fig. 3.**
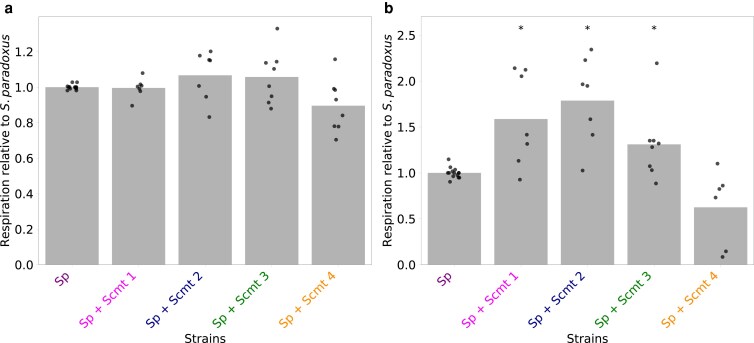
*S. cerevisiae* mitotypes boost respiration in the *S. paradoxus* nuclear background at 39°C. In each panel, the *y*-axis reports measurements of the reduction of 1-(4,5-dimethylthiazol-2-yl)-3,5-diphenyltetrazolium bromide after 24 h of growth of a given strain in liquid culture with glucose as the carbon source at one temperature, normalized to the respective measurement wild-type *S. paradoxus*. In a given column, each point reports results from one biological replicate and the bar height reports the average. The *x*-axis denotes strain genotypes: wild-type *S. paradoxus* (Sp) or cybrids each harboring the mitochondrial genome from a *S. cerevisiae* donor (Scmt) labeled as in Fig. 1. a) 28°C; b) 39°C. *, one-sample Wilcoxon *P* < 0.05. Raw data and statistical analyses are reported in Supplementary Table 2h and i.

As a more direct measure of metabolic effects driven by *S. cerevisiae* mitochondria DNA, we established an assay of reduction of the colorimetric substrate 1-(4,5-dimethylthiazol-2-yl)-3,5-diphenyltetrazolium bromide (MTT) in glucose medium. This signal serves as a reporter of activities of the electron transport chain, particularly succinate dehydrogenase (Slater et al. 1963; Sánchez and Königsberg 2006; Jain et al. 2018) (though see Shoemaker et al. (2004) and Peng et al. (2005)), and thus is informative as a marker of respiration. A first set of observations revealed no detectable impact of mitotype on MTT reduction during culture in liquid glucose medium at 28 °C in the *S. paradoxus* background (Fig. 3a and Supplementary Fig. 5a). We reasoned that the metabolic effect of *S. cerevisiae* mitotypes might be better resolved at higher temperatures, in which yeast cells respire particularly avidly (Rikhvanov et al. 2001 ). Consistent with this notion, in culture at 39 °C in glucose medium, we observed increased MTT reduction in all cybrids except for that harboring *S. cerevisiae* Y55 mitochondrial DNA, relative to wild-type Z1 *S. paradoxus* with its endogenous mitochondrial genome (Fig. 3b and Supplementary Fig. 5b). Together, these results established that in the *S. paradoxus* background, *S. cerevisiae* mitotypes can be sufficient for increased growth and metabolic behaviors that are signatures of respiration.

### *S. cerevisiae* mitotypes boost thermotolerance in *S. paradoxus*

We now turned to high-temperature growth proper, as an additional phenotype that we hypothesized would be modulated by mitotype in our yeast species comparison. We measured cell density during growth in liquid glucose medium at 39°C, for each of our cybrid strains harboring *S. cerevisiae* mitochondrial DNA in Z1 *S. paradoxus*. Results revealed a 10% to 70% improvement in accumulated biomass in this assay, relative to wild-type *S. paradoxus,* in all cybrids except the one for which *S. cerevisiae* Y55 was the mitochondrial donor (Fig. 2a). In an expanded panel of cybrids using additional *S. cerevisiae* mitotype donors, a boost in thermotolerance was also apparent in all cases (Supplementary Fig. 6). Thus, *S. cerevisiae* mitotypes were sufficient to confer thermotolerance benefits, in some cases sizeable ones, in *S. paradoxus*. Controls established that *S. cerevisiae* mitotypes had phenotypic effects in a given *S. cerevisiae* background mirroring those in *S. paradoxus*, though no *S. cerevisiae* cybrid exceeded the thermotolerance of the wild type of this species (Supplementary Fig. 7). Interestingly, after 24 h of incubation at 39°C, cultures of cybrids in the *S. paradoxus* background harbored very few viable cells (Supplementary Fig. 8). We conclude that in such cybrids, *S. cerevisiae* mitochondrial DNAs enable additional cell divisions during high-temperature exposure, after which failures of factors encoded by the *S. paradoxus* nuclear genome ultimately prevent further growth and kill cells outright.

Thermotolerance phenotypes as they varied across our panel of *S. paradoxus* cybrids correlated tightly with the respiratory behaviors we had documented in assays of growth in nonfermentable carbon sources in these strains (Fig. 2a and b) and MTT reduction (Fig. 3). That is, among *S. paradoxus* cybrids, those performing best in respiratory contexts were the most thermotolerant, and the worst in respiratory assays grew the worst at high temperature. These results are most consistent with a causal relationship between the traits, whereby the *S. cerevisiae* mitotypes that foster better thermotolerance do it by improving respiration—a metabolic underpinning for the capacity to grow under high-temperature challenge.

### Antagonistic pleiotropy by *S. cerevisiae* mitotypes in temperate conditions and ethanol

Previous studies using interspecies hybrids have reported a defect in cold temperatures attributable to *S. cerevisiae* mitotypes relative to those of *S. paradoxus* or other relatives (Baker et al. 2019; Li et al. 2019; Hewitt et al. 2020). To test whether such effects would be recapitulated in purebred backgrounds, in Z1 *S. paradoxus* derivatives carrying *S. cerevisiae* mitochondrial DNA, we assayed the carrying capacity after culture 23°C in liquid glucose medium, for comparison against a 28°C control and the high-temperature condition (39 °C), also with glucose as a carbon source. The results revealed compromised cell density at 23°C in the cybrids that performed the best at high temperature (Fig. 2a). This set up an almost perfect anticorrelation between 23°C growth and thermotolerance across strains (Fig. 2c), mirroring the observation we had made with respiratory behaviors (Figs. 2 and 3). We conclude that *S. cerevisiae* mitotypes have specialized to heat stress at the expense of performance at lower temperatures, suggestive of fine-tuning by evolution of the temperature optima of respiratory reactions encoded by the mitochondrial genome.

We also noted an anticorrelation across our cybrids between thermotolerance and cell density in glucose at 28°C: at the latter temperature, the most thermotolerant strains harboring *S. cerevisiae* DNA performed the worst (Fig. 2a). To explore potential drivers of this pattern, we also assayed cell density at 28°C in medium with ethanol as the carbon source, in which we observed most cybrids carrying *S. cerevisiae* mitochondrial DNA to be at a disadvantage (Fig. 2a). Growth patterns among strains at 28°C and in ethanol were robustly correlated (Fig. 2d), suggestive of a mechanistic relationship between them, as expected given that *Saccharomyces* species catabolize ethanol late in the growth cycle after having secreted it (Piskur et al. 2006). In summary, based on our growth and respiration profiles, we conclude that *S. cerevisiae* mitochondrial genomes are drivers of a phenotypic syndrome that includes advantages in most facets of respiration as well as high-temperature growth, and disadvantages at cooler temperatures and during ethanol utilization.

### Positive epistasis between mitochondria and nuclear genomes in terms of thermotolerance

**Fig. 4.**
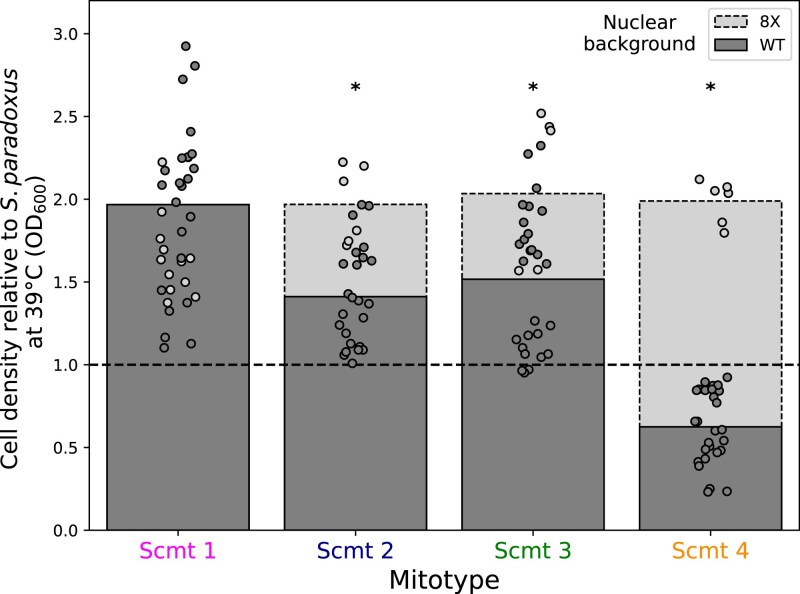
Epistasis between nuclear and mitochondrial *Saccharomyces* species variants that impact thermotolerance. In a given column, each color reports results from 24 h of growth at 39°C, with glucose as the carbon source, of a strain of *S. paradoxus* with (8X) or without (WT) 8 thermotolerance loci from *S. cerevisiae* that harbored an *S. cerevisiae* mitotype. The *y*-axis reports the cell density of the indicated strain normalized to that of wild-type *S. paradoxus* with its native mitotype. Strain genotypes are as in Fig. 1. Each point reports one replicate (*n* ≥ 6). *, the interaction term from a two-factor ANOVA with mitotype and nuclear genotype as factors was significant at *P* < 0.01. Raw growth measurements and statistical analyses are reported in Supplementary Table 2a and j.

During the evolution of *S. cerevisiae* as a species, the pro-thermotolerance mitotypes we study here would have arisen alongside changes in nuclear genes which could modify their effects. We sought to shed light on these relationships by modeling them in the *S. paradoxus* background. To this end, we introduced *S. cerevisiae* mitochondrial genomes into a derivative of *S. paradoxus* Z1 that also harbored the alleles from the DBVPG1373 Dutch soil strain of *S. cerevisiae* at eight nuclear loci which contribute to thermotolerance (*AFG2*, *APC1*, *CEP3*, *DYN1*, *ESP1*, *MYO1*, *SCC2*, and *TAF2*, most of which encode cell division factors; AlZaben et al. 2021). In assays of cell density at 39°C, the combination of *S. cerevisiae* alleles in the nuclear and mitochondrial genomes together conferred a 2-fold improvement relative to wild-type *S. paradoxus* (Fig. 4, gray bars). This represented an increase in cell divisions early in the treatment, as essentially no cybrid cells harboring *S. cerevisiae* nuclear alleles maintained viability after 24 h at 39°C (Supplementary Fig. 9), paralleling results from cybrids in the wild-type *S. paradoxus* nuclear background (Supplementary Fig. 8). Advantages in cell density at 39°C manifested regardless of the origin of the mitotype: mitochondrial DNA from across our panel of *S. cerevisiae* strain donors yielded nearly identical high-temperature growth in *S. paradoxus* in the presence of the nuclear thermotolerance genes from *S. cerevisiae,* including the Y55 mitotype that had compromised the phenotype in wild-type *S. paradoxus* (Figs. 2a and 4). Thus, the latter incompatibility was resolved by *S. cerevisiae* alleles at our focal nuclear genes.

Also evident from our nuclear-mitochondrial transgenic strain panel was a pattern of positive epistasis. That is, the impact of each *S. cerevisiae* mitotype on thermotolerance was greater in the *S. paradoxus* derivative also harboring nuclear alleles from *S. cerevisiae* than in otherwise wild-type *S. paradoxus* (Fig. 4; compare gray and black bars). Mitochondrial DNA from distinct *S. cerevisiae* donors, including Y55, all achieved peak performance at high temperature in the presence of *S. cerevisiae* nuclear genes; no such dependence was detectable in 28°C cultures (Supplementary Fig. 10). That said, the *S. cerevisiae* mitotype that promoted thermotolerance most strongly on its own in the *S. paradoxus* background, from DBVPG1373, exhibited almost no benefit from the addition of DBVPG1373 alleles of nuclear genes (Fig. 4, magenta). Relative to this transgenic, strains harboring other *S. cerevisiae* mitotypes, which had less impact on their own, achieved the same cell density at 39°C in the presence of DBVPG1373 nuclear alleles (Fig. 4, blue, green, and orange). These results are most consistent with a ceiling beyond which any *S. cerevisiae* alleles of the mitochondrial genome, even when combined with *S. cerevisiae* alleles of our eight focal nuclear genes, are unable to improve thermotolerance, in the face of defects accruing from other *S. paradoxus* loci. Nonetheless, our data make clear that pro-thermotolerance alleles of mitochondrial DNAs and nuclear genes from *S. cerevisiae* together contribute to the phenotype in a manner that exceeds the sum of their parts.

## Discussion

A key goal of comparative genetics is to understand how evolution builds traits over long timescales. In this work, we have leveraged tractable *Saccharomyces* as a testbed for the study of interspecies trait genetics and evolution focused on the mitochondrial genome. Given the evidence that *S. cerevisiae* thermotolerance is the product of positive selection (Weiss et al. 2018; Abrams et al. 2021), it serves as a powerful model of the evolutionary genetics of adaptation over deep divergences. Our findings complement previous studies of mitotype effects in this system (Baker et al. 2019; Li et al. 2019; Hewitt et al. 2020) by tracing the phenotypes conferred by *S. cerevisiae* mitochondrial alleles in purebred backgrounds, including their genetic dependencies.

Our results shed new light on the genetic architecture and evolution of yeast thermotolerance. Our finding of a 70% boost in the trait by an *S. cerevisiae* mitotype outstrips the effect of any one of the nuclear loci previously reported in this system (Weiss et al. 2018). Sequence analyses revealed amino acid alleles unique to, and largely conserved in, *S. cerevisiae* in the *COX1* gene that represent candidate determinants of the mitochondrial effects we study here (Supplementary Fig. 11). But we have also seen that phenotypic impacts differ among *S. cerevisiae* mitotypes, consistent with the extensive precedent for polymorphism among mitochondrial genomes and their effects on human disease (Wallace 2015) as well as traits in other animal models (Mossman et al. 2016a; Mossman, Biancani, et al. 2016b; Patel et al. 2016; Tsai and St. John 2016; Camus et al. 2017; Camus and Dowling 2018; Mossman et al. 2019; Camus et al. 2020; Salminen and Vale 2020; Anderson et al. 2022; Brand et al. 2024; Serrano et al. 2024) and plants (Dubrovina and Kiselev 2016; Adhikari et al. 2019; Jo et al. 2019). Furthermore, none of our mitochondrial manipulations reconstituted more than a fraction of the phenotype of the *S. cerevisiae* wild type in the *S. paradoxus* background, congruent with the very modest effects of *S. cerevisiae* nuclear alleles known to be adaptive in this system (Weiss et al. 2018; AlZaben et al. 2021). Thus, our findings deepen the emerging picture of a highly complex architecture for yeast thermotolerance, with mitochondrial variants and many nuclear loci of small to modest effect coming together along the *S. cerevisiae* lineage to build the adaptation.

Our data also make clear that alongside largely beneficial marginal effects, all but one of the *S. cerevisiae* mitotypes we study are even more advantageous at high temperature in the context of pro-thermotolerance nuclear alleles (the exception being the mitotype from Dutch soil isolate DBVPG1373). To date, such evidence for positive epistasis has been at a premium in the literature describing adaptive loci from the wild (Nelson and Grishin 2018; Syenina et al. 2020; Stern et al. 2022). This may be in part owing to a heavier emphasis in the field on negative epistasis in the evolutionarily difficult repacking of a given protein interior to achieve fitness (Weinreich et al. 2005; Starr and Thornton 2016), which could be more constrained than combinations of adaptive alleles of multiple proteins. And negative epistasis has been rife between mitochondrial and nuclear variants in analysis of traits that are not likely to promote fitness, including reproductive barriers and aging and disease (Ellison and Burton 2008; Arnqvist et al. 2010; Tranah 2011; Joseph et al. 2013; Lovell et al. 2015; Roux et al. 2016; Loewen and Ganetzky 2018; Wolters et al. 2018; Sujkowski et al. 2019; Nguyen et al. 2020, 2023; Bushel et al. 2022; Biot-Pelletier et al. 2023; Ibrahim et al. 2024). But even in the unlinked nuclear genes contributing to yeast thermotolerance, negative epistasis is the rule rather than the exception (AlZaben et al. 2021). As such, our data point to pro-thermotolerance mitoalleles as uniquely subject to positive epistasis with the nuclear genome in this adaptive system. Under one compelling model, these mitochondrial variants may have been especially easily accessible (Liao et al. 2022) and beneficial to populations during the evolution of *S. cerevisiae* thermotolerance, representing the so-called low-hanging fruit (Lenski 2017) of the adaptive process.

Though our data leave open the precise mechanism for such benefits, we propose that *S. cerevisiae* mitotypes produce extra energy and metabolites (Malecki et al. 2020) during thermal stress via enhanced respiration, relative to the putatively ancestral phenotypic program attributable to mitochondria from *S. paradoxus*. If so, an excess of basic building blocks would support the capacity of *S. cerevisiae* nuclear genes to confer their effects. The nuclear loci we study here govern housekeeping functions, namely cell division and transcription/translation, and some may prove to contribute more than others to the epistatic relationship with the mitochondrial genome.

Meanwhile, the growth defects we have seen by cybrids at lower temperatures dovetail with results from studies of *S. cerevisiae* nuclear thermotolerance loci and those using interspecies backgrounds (Baker et al. 2019; Li et al. 2019; Hewitt et al. 2020; AlZaben et al. 2021). As a general rule, evolutionary tradeoffs are routine in the adaptation literature, from prokaryotes (Abram et al. 2021; Backman et al. 2024; Dalldorf et al. 2024) to animals (Ou et al. 2020; Ackermans 2023; Metzler et al. 2023) and plants (Gao et al. 2022; He et al. 2022; Kurepa and Smalle 2023). For yeast in particular, the exact mechanism of antagonistic pleiotropy exhibited by thermotolerance loci in cooler conditions remains unclear. Exciting recent work has revealed that *S. cerevisiae* proteins, including those encoded by mitochondria, are more thermostable than those of sister species, establishing the likely biochemical underpinning of the trait (Walunjkar et al. 2025). But it may not follow that the *S. cerevisiae* alleles of the loci we study here compromise protein structure per se at low temperatures (Yang et al. 2015; Cui et al. 2024). Amino acid changes unique to *S. cerevisiae* could well have tuned the temperature optima for the function of many enzymes, as appears to be the case for the thermotolerance gene *DYN1* (Hong et al. 2016). Further work will be required to pinpoint exactly what biochemical mechanisms *S. cerevisiae* gave up as it acquired pro-thermotolerance alleles. As they stand, however, the genetics of this system help tell a story of a complex balancing act by evolution along the *S. cerevisiae* lineage, as nuclear and mitochondrial variants were assembled to convert a thermosensitive ancestor to a high-temperature specialist.

## Supplementary Material

iyaf233_Supplementary_Data

## Data Availability

The authors affirm that all data necessary for confirming the conclusion of the article are present within the article, figures, and tables. Strains are available upon request. Supplemental material available at GENETICS online.
