# Supplementary material for: The role of mitotype variation and positive epistasis in trait differences between Saccharomyces species
Source: Genetics. 2025 Oct 27;232(1):iyaf233. doi: 10.1093/genetics/iyaf233 (PMC12774845; doi:10.1093/genetics/iyaf233)
Supplement: iyaf233_Supplementary_Data [file iyaf233_supplementary_data.zip › Supplementary_Material_Captions_GENETICS-2025-308167R1.docx]

Supplementary figure captions

**Supplementary Figure 1. Stark growth differences between *S. cerevisiae* and *S. paradoxus* at high temperature.** In a given panel, the *y*-axis reports cell density after 24 hours of growth in liquid culture of a wild-type homozygous diploid *Saccharomyces* strain in one temperature condition, with glucose as the carbon source. Points reports technical replicates (*n* = 3) and bar heights report means. Sc1, Sc2, Sc3 and Sc4 denote *S. cerevisiae* DBVPG1373, YPS128, DBVPG6044 and Y55, respectively. Sp1, Sp2, Sp3 and Sp4 denote *S. paradoxus* KPN3828, UFRJ50816, A4 and Z1, respectively. **(A)** 28°C; **(B)** 39°C. *, two-tailed Mann-Whitney *p* < 0.05. Raw growth measurements and statistical analyses are reported in Supplementary Table 2K.

**Supplementary Figure 2. Wild-type strain and species variation in growth on glycerol.** In a given panel, each bar reports results from the indicated wild-type *S. cerevisiae* or *S. paradoxus* strain grown in liquid culture with glycerol as the carbon source, normalized with respect to a standard reference-strain control, from (Warringer et al. 2011). The *y*-axis reports relative growth rate **(A)** and efficiency **(B)**. *, two-tailed Mann-Whitney *p* < 0.05. Raw growth measurements and statistical analyses are reported in Supplementary Table 2L to 2N.

**Supplementary Figure 3. Condition-dependent growth across *Saccharomyces* species and cybrids.** Data are as in Figure 2A except that wild-type *S. cerevisiae* (DBVPG1373) is included. Raw growth measurements and statistical analyses are reported in Supplementary Table 2A to 2G.

**Supplementary Figure 4. No detectable differences in glycogen and trehalose storage in *S. paradoxus* cybrids.** In a given panel, each bar reports energy storage by cells of the indicated strain after growth with glycerol as the carbon source at 28°C, either glycogen (A) or trehalose (B). Each dot denotes one biological or technical replicate. Labels on the *x*-axis are as in Figure 2A of the main text. Raw trehalose and glycogen measurements and statistical analyses are reported in Supplementary Table 2P and 2Q.

**Supplementary Figure 5. *S. cerevisiae* mitotypes boost respiration in the *S. paradoxus* nuclear background at 39°C with addition of *S. cerevisiae*.** Data are as in Figure 3 except that wild-type *S. cerevisiae* (DBVPG1373) is included. Raw MTT measurements and statistical analyses are reported in Supplementary Table 2H and 2I.

**Supplementary Figure 6. *S. cerevisiae* mitotypes are sufficient to boost thermotolerance in *S. paradoxus*.** Each column reports results from *S. paradoxus* harboring mitochondrial DNA from a *S. cerevisiae* strain. The *y*-axis reports cell density after 24 hours of growth at 39°C of a cybrid normalized to that of the wild-type *S. paradoxus*, which is shown as the dashed line. *, two-tailed Mann-Whitney *p* < 0.05. Bar height reports average across replicates, and each dot is one technical or biological replicate. Scmt 1 to Scmt 8 denote mitotypes from Sc 1 to Sc 8 in Figure 1. Raw growth measurements and statistical analyses are reported in Supplementary Table 2A and 2O.

**Supplementary Figure 7. Effects of *S. cerevisiae* mitotypes in *S. cerevisiae* on thermotolerance echo those in *S. paradoxus*.** Each column reports results from *S. cerevisiae* harboring mitochondrial DNA from a *S. cerevisiae* strain. The *y*-axis reports cell density after 24 hours of growth at 39°C. *, two-tailed Mann-Whitney *p* < 0.05. Bar height reports average across replicates, and each dot is one technical or biological replicate. Labels on the *x*-axis are as in Figure 2A. Raw growth measurements and statistical analyses are reported in Supplementary Table 2A and 2J.

**Supplementary Figure 8. Chronic 39°C treatment is lethal to *S. paradoxus* and its cybrids.** Shown are results of plating of the indicated strains after 24 hours of culture in one temperature condition, with glucose as the carbon source, followed by incubation of plates at 28°C for two days. Strains were wild-type *S. cerevisiae* (Sc), wild-type *S. paradoxus* (Sp), or a cybrid in the *S. paradoxus* background harboring the mitochondrial genome from a *S. cerevisiae* donor (Scmt) labeled as in Figure 1 of the main text.

**Supplementary Figure 9. Chronic 39°C treatment is lethal to *S. paradoxus* harboring *S. cerevisiae* nuclear thermotolerance loci and to its cybrids.** Labels are as in Supplementary Figure 7 except that the strains were wild-type *S. cerevisiae* (Sc), wild-type *S. paradoxus* (Sp), *S. paradoxus* harboring eight nuclear loci from *S. cerevisiae* (8X), or a cybrid in the 8X background harboring the mitochondrial genome from a *S. cerevisiae* donor (Scmt) labeled as in Figure 1 of the main text.

**Supplementary Figure 10. No detectable** **epistasis between nuclear and mitochondrial *Saccharomyces* species variants that impact 28°C growth.** Data are as Figure 4 of the main text except that cell densities were measured after 24 hours of growth at 28°C. Raw growth measurements and statistical analyses are reported in Supplementary Table 2A and 2J.

**Supplementary Figure 11. A multiple sequence alignment analysis shows highly diverged amino acid variants in *COX1* between *S. paradoxus* and *S. cerevisiae*.** At top, each line reports one *COX1* amino acid sequence of *S. cerevisiae* (737 strains) and *S. paradoxus* (27 strains) as denoted on the left. At bottom, bar heights in each panel report the frequency of the indicated amino acid allele in the indicated species. Not shown are infrequent alleles in *S. cerevisia*e., namely N (0.3%) and T (0.1%) at position 55 and premature stop codons (0.3%) at position 58. Mitochondrial genome accessions are cited in the Materials and Methods section.

Supplementary table captions

**Supplementary Table 1. Strains used in this work.**

**Supplementary Table 2. Growth data and statistics.** D1373, YPS128, D6044 and Y55 indicate *S. cerevisiae* strains DBVPG1373, YPS128, DBVPG6044 and Y55, respectively; Sp, *S. paradoxus* Z1. Supplementary Table 2A contains all OD_600_ measurements of each strain at 39°C in standard glucose medium. Supplementary Table 2B reports the statistical analysis of the OD_600_ measurements of the cybrids in **(A)** compared to wild-type *S. paradoxus* or the *S. paradoxus* harboring *S. cerevisiae* nuclear loci (8X) by Mann-Whitney U test. Supplementary Table 2C contains all OD_600_ measurements of each strain at 28°C in standard glucose medium. Supplementary Table 2D contains all OD_600_ measurements of each strain at 28°C in medium with glycerol as a carbon source. Supplementary Table 2E contains all OD_600_ measurements of each strain at 28°C in medium with ethanol as a carbon source. Supplementary Table 2F contains all OD_600_ measurements of each strain at 23°C in standard glucose medium. Supplementary Table 2G reports the Spearman correlation coefficients and respective *p*-values of Figures 2B, 2C and 2D. Table 2H reports the raw readouts of the MTT assay at 28°C and 39°C. Supplementary Table 2I reports the *p*-values of the normalized MTT measurements by one-sample Wilcoxon with a hypothesized value of 1. Supplementary Tabel 2J reports the *p*-values of two-way ANOVA of the impact of the interaction between *S. cerevisiae* mitotypes and the 8 *S. cerevisiae* loci in the *S. paradoxus* background on high temperature growth (Figure 4). Supplementary Table 2K reports the OD_600_ measurements of wild-type *S. cerevisiae* and *S. paradoxus* strains at 28°C and 39°C in standard glucose medium with the statistical analysis (Supplementary Figure 1). Supplementary Table 2L and 2M report the growth efficiency and rate in glycerol as the carbon source from (Warringer et al. 2011). Supplementary Table 2N reports the *p*-values of glycerol growth efficiency and rate of the wild-type *S. cerevisiae* and *S. paradoxus* strains by two-tailed Mann-Whitney U test (Warringer et al. 2011) (Supplementary Figure 2). Supplementary Table 2O reports the *p*-values of Supplementary Figure 5 by two-tailed Mann-Whitney U test. Supplementary Table 2P and 2Q report the raw readouts of trehalose and glycogen assays and *p*-values by two-tailed Mann-Whitney U test, respectively (Supplementary Figure 10).
