# Supplementary figures and images for: The role of mitotype variation and positive epistasis in trait differences between Saccharomyces species
Source: Genetics. 2025 Oct 27;232(1):iyaf233. doi: 10.1093/genetics/iyaf233 (PMC12774845; doi:10.1093/genetics/iyaf233)

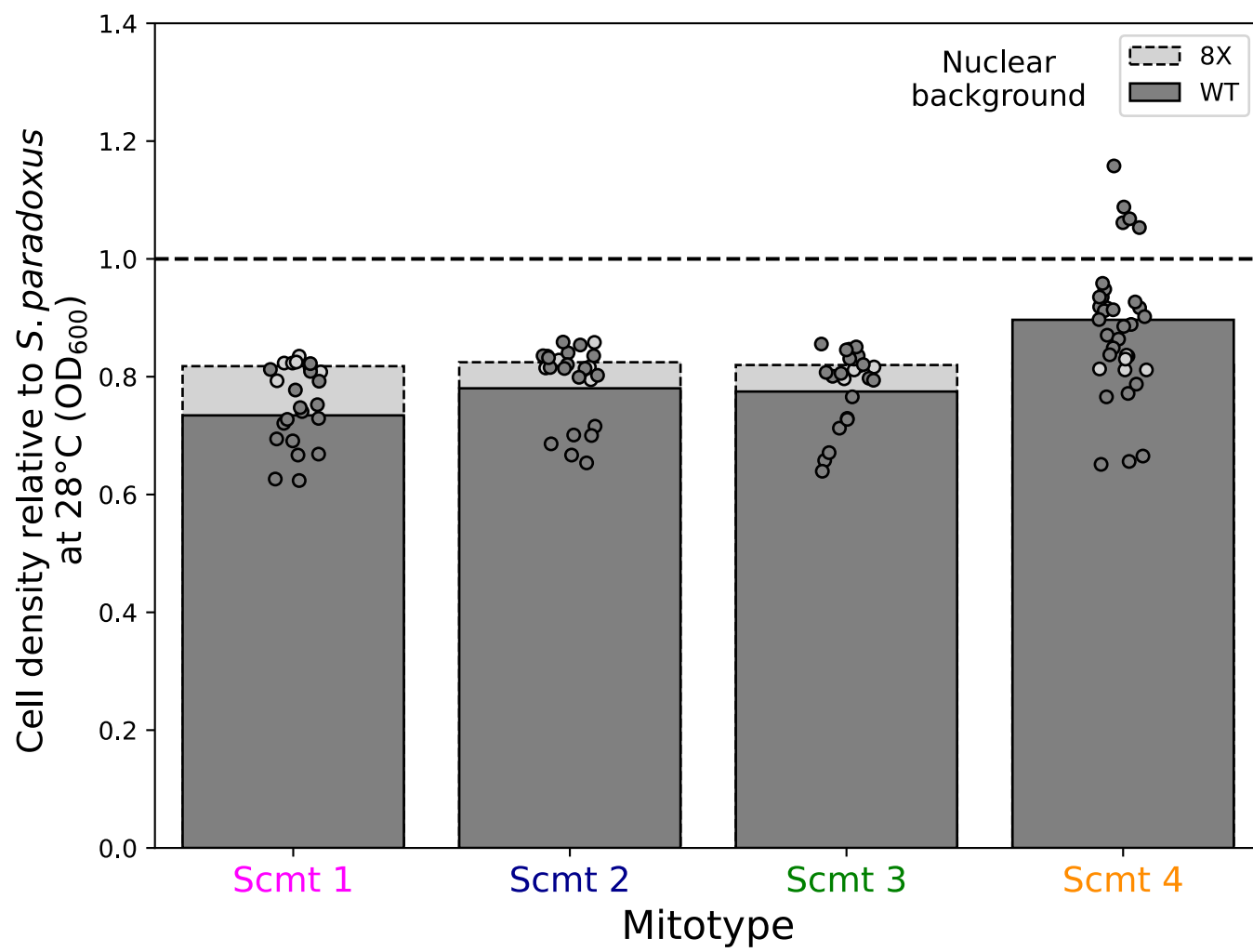

Supplement: iyaf233_Supplementary_Data [file iyaf233_supplementary_data.zip › Supplementary_Figure_10_GENETICS-2025-308167R1.pdf]

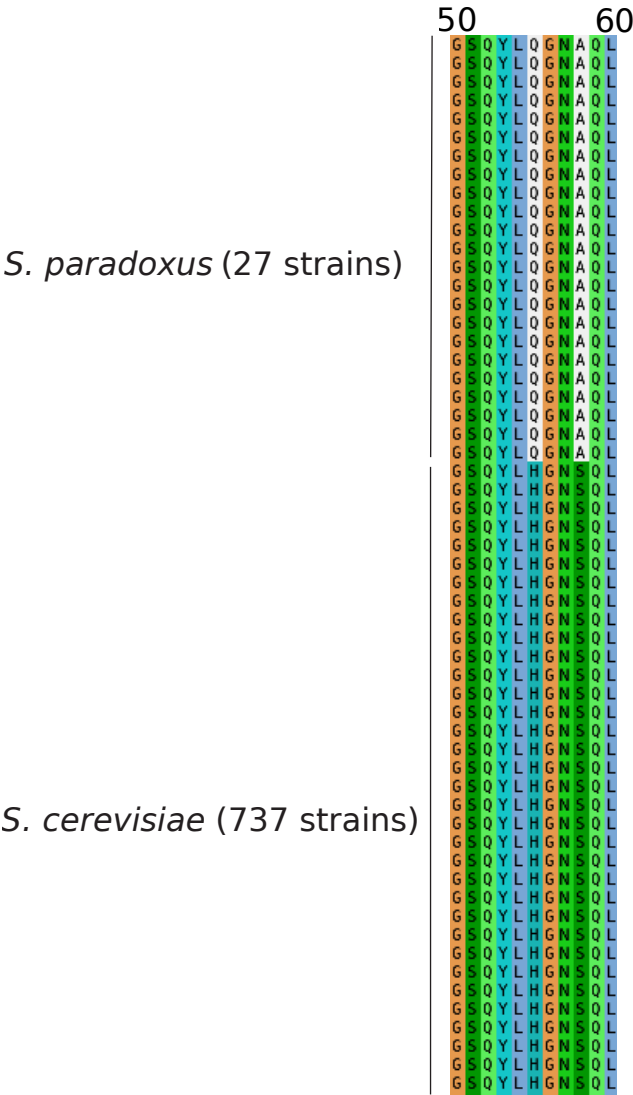

Supplement: iyaf233_Supplementary_Data [file iyaf233_supplementary_data.zip › Supplementary_Figure_11_GENETICS-2025-308167R1.pdf]

(A)

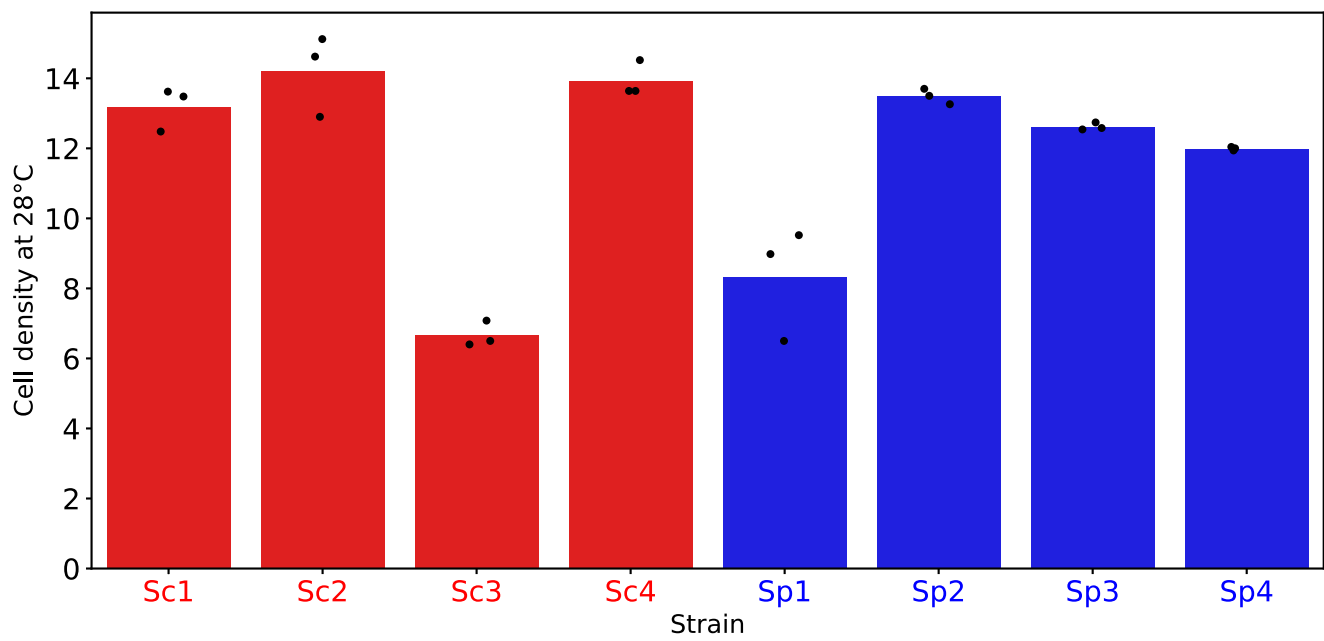

(B)

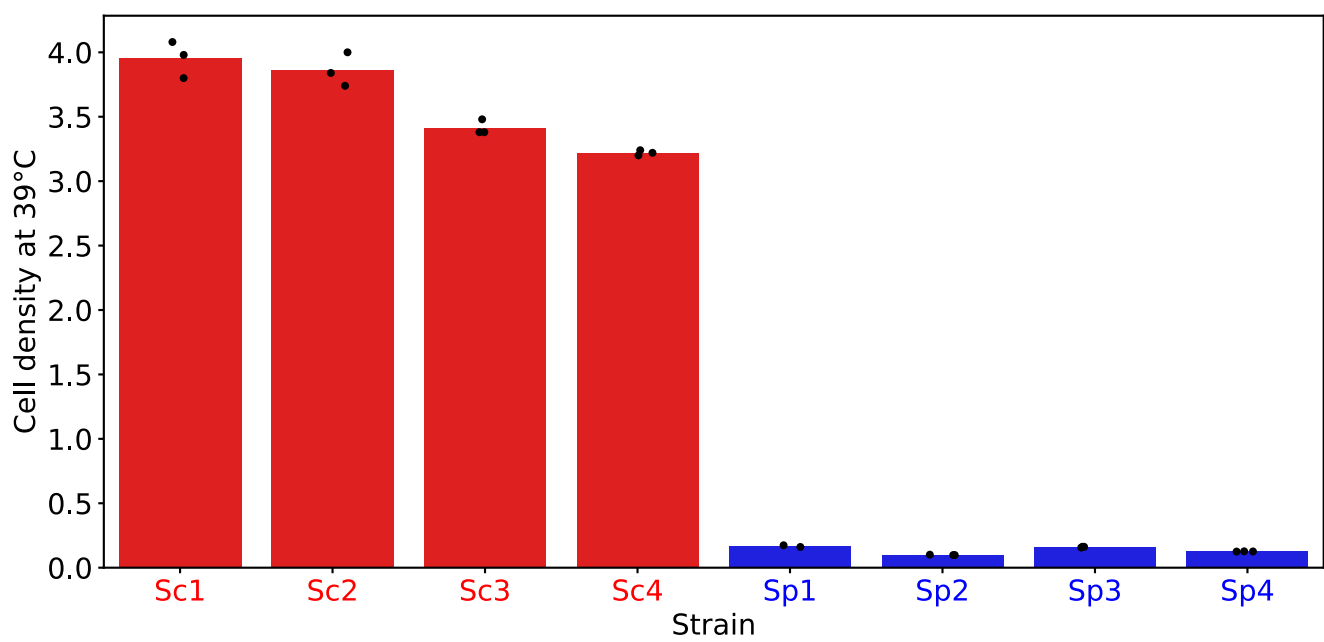

Supplement: iyaf233_Supplementary_Data [file iyaf233_supplementary_data.zip › Supplementary_Figure_1_GENETICS-2025-308167R1.pdf]

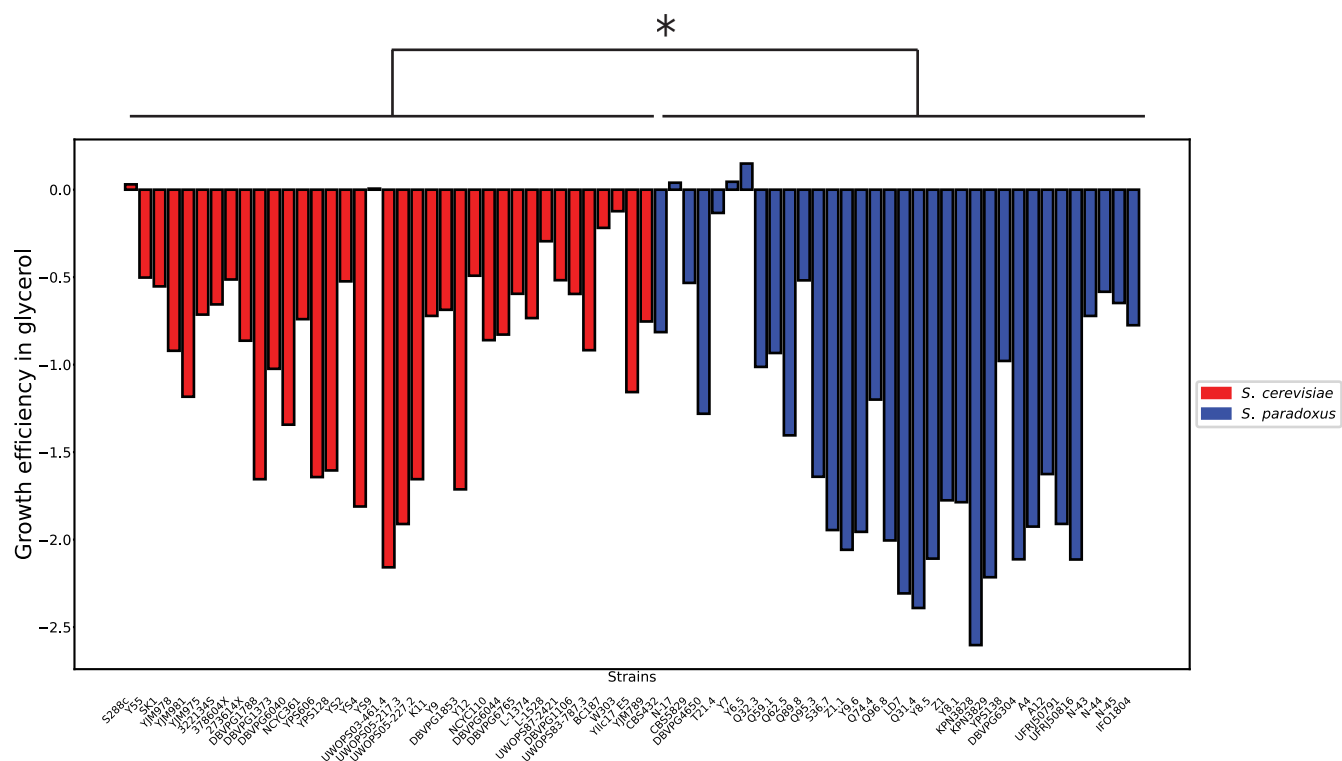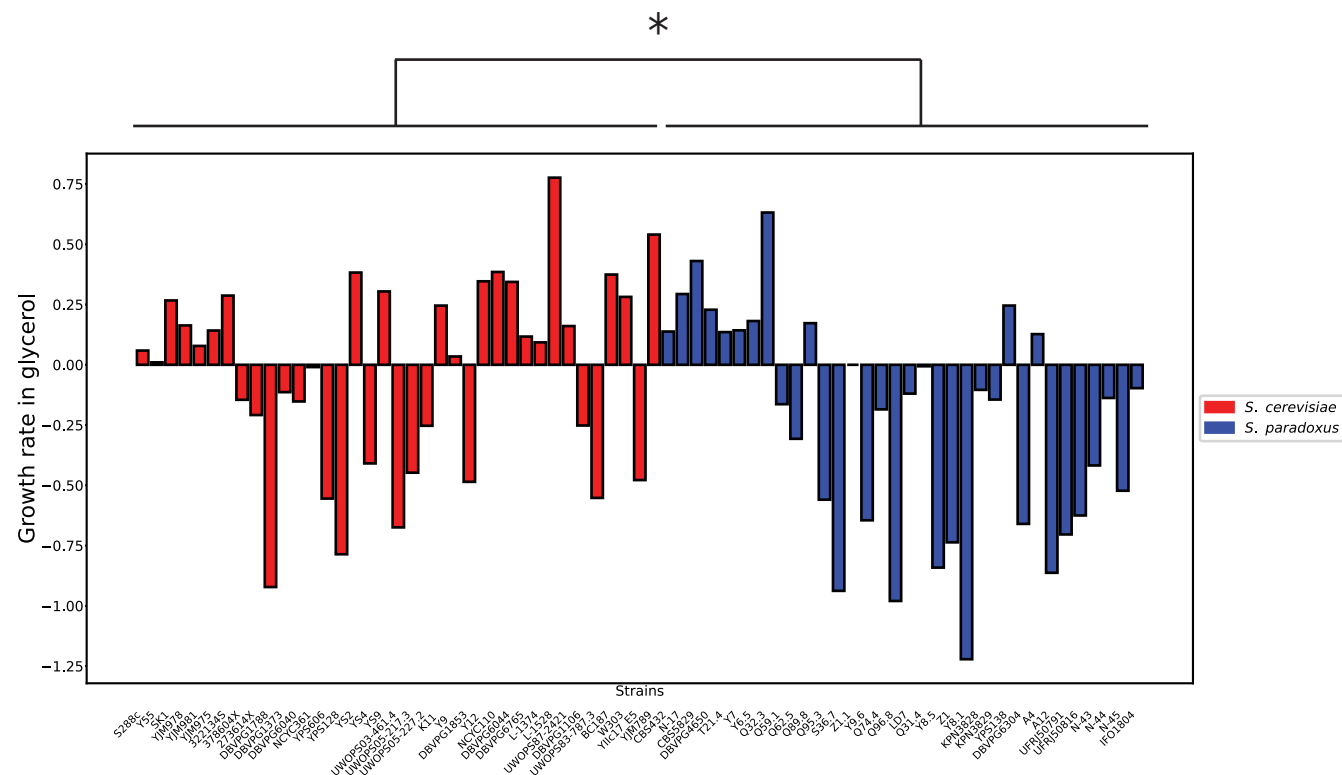

Supplement: iyaf233_Supplementary_Data [file iyaf233_supplementary_data.zip › Supplementary_Figure_2_GENETICS-2025-308167R1.pdf]

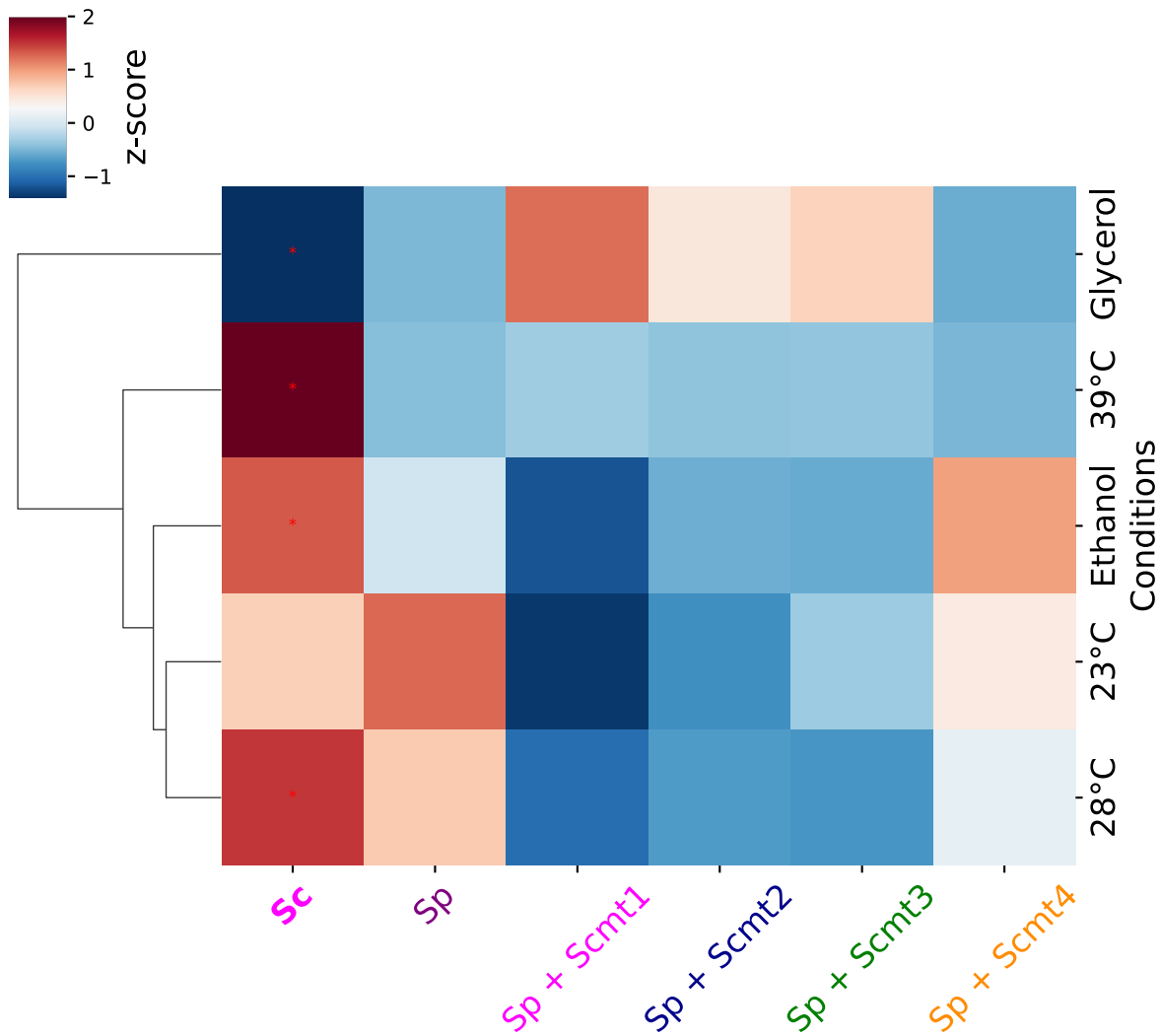

Supplement: iyaf233_Supplementary_Data [file iyaf233_supplementary_data.zip › Supplementary_Figure_3_GENETICS-2025-308167R1.pdf]

(A)

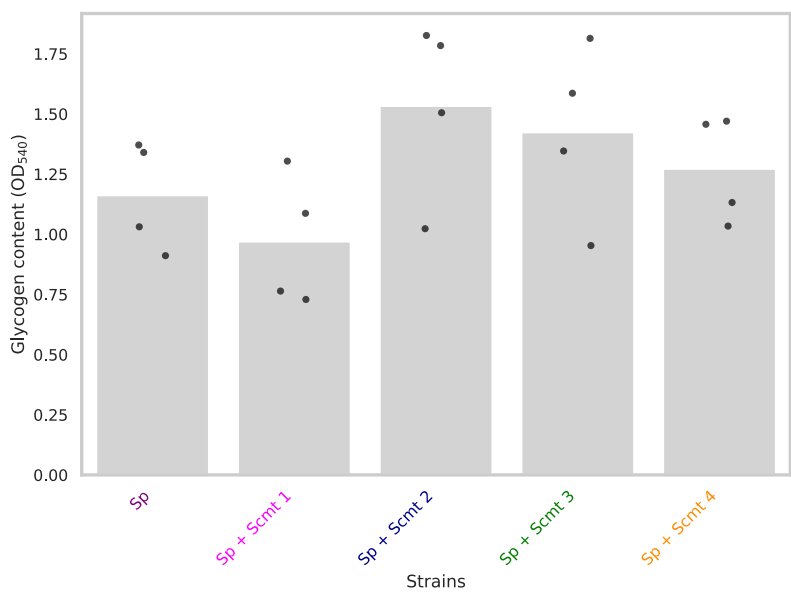

(B)

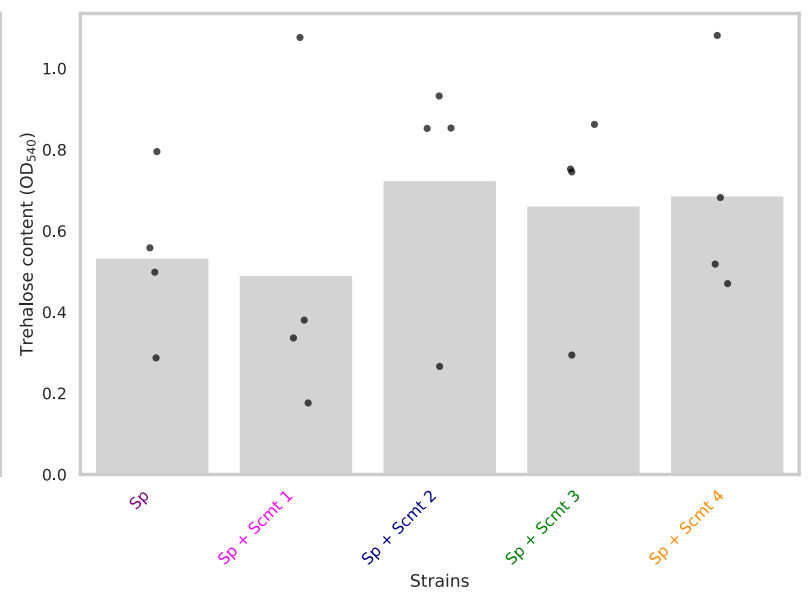

Supplement: iyaf233_Supplementary_Data [file iyaf233_supplementary_data.zip › Supplementary_Figure_4_GENETICS-2025-308167R1.pdf]

(A)

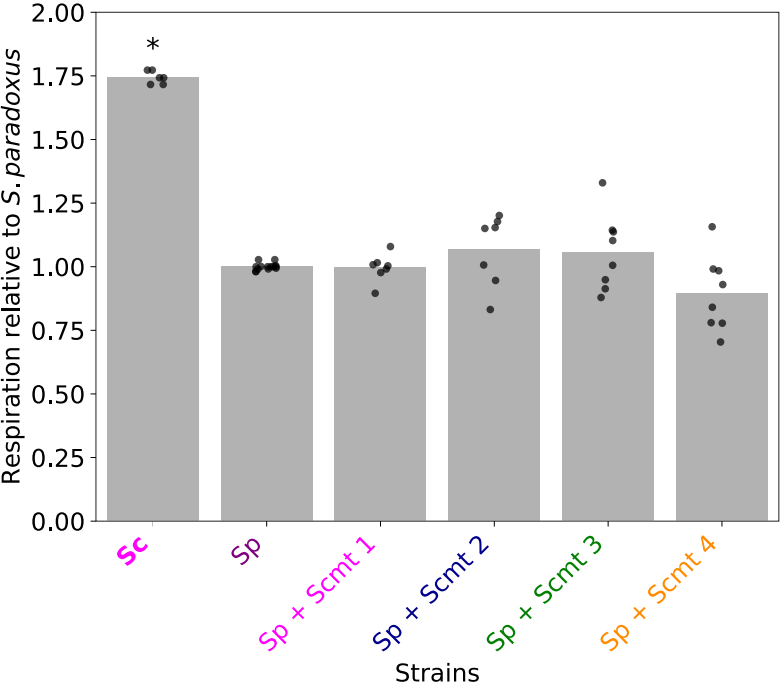

(B)

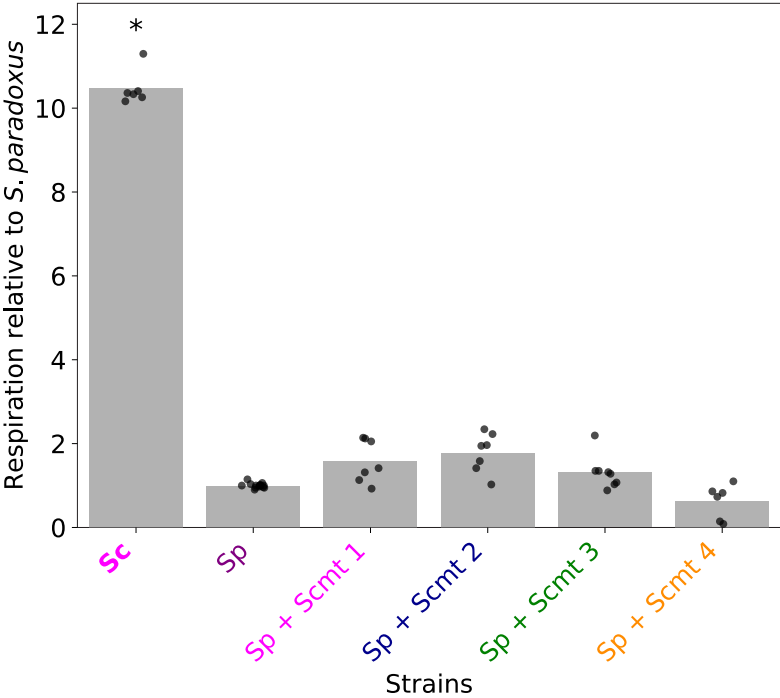

Supplement: iyaf233_Supplementary_Data [file iyaf233_supplementary_data.zip › Supplementary_Figure_5_GENETICS-2025-308167R1.pdf]

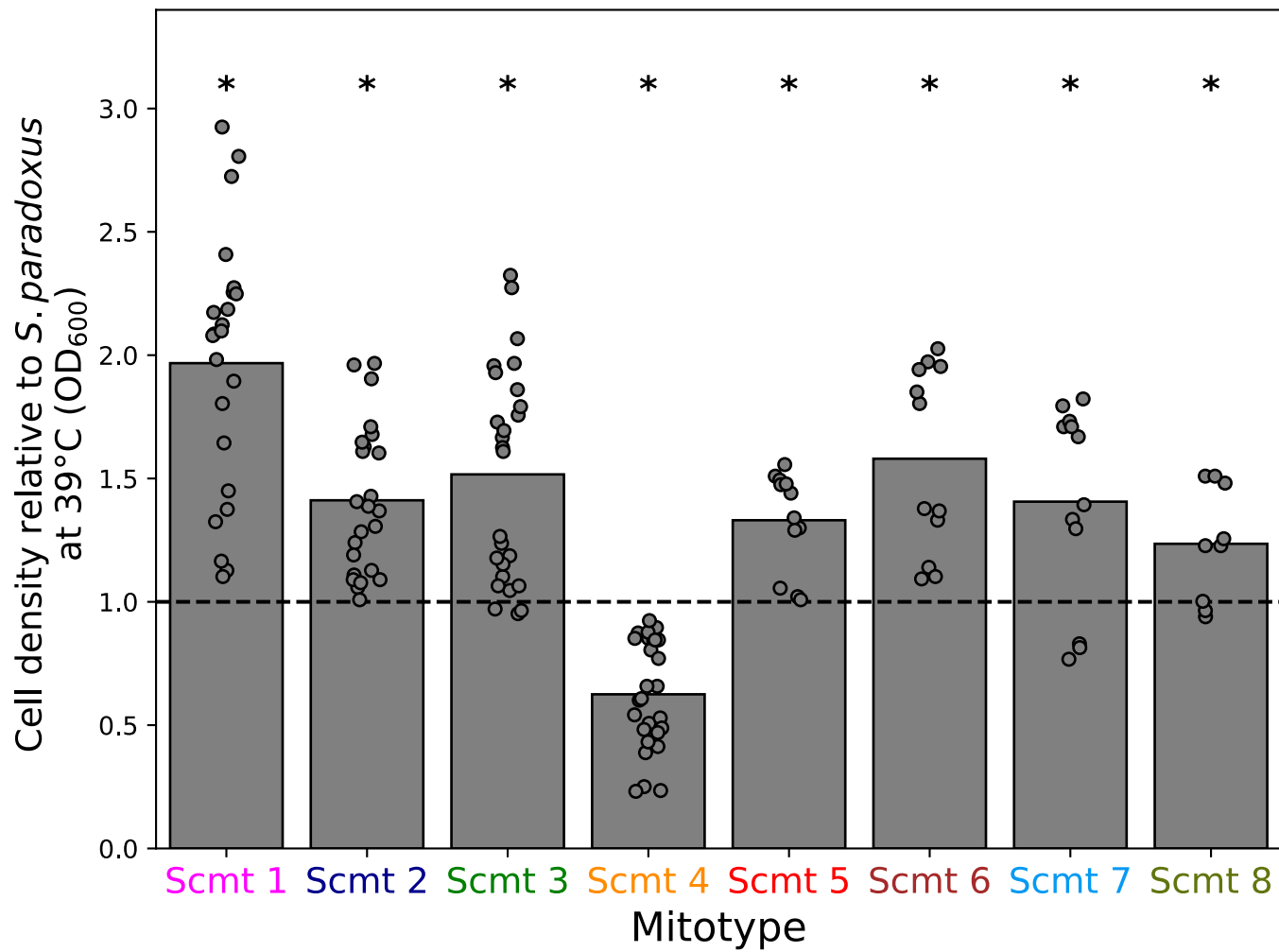

Supplement: iyaf233_Supplementary_Data [file iyaf233_supplementary_data.zip › Supplementary_Figure_6_GENETICS-2025-308167R1.pdf]

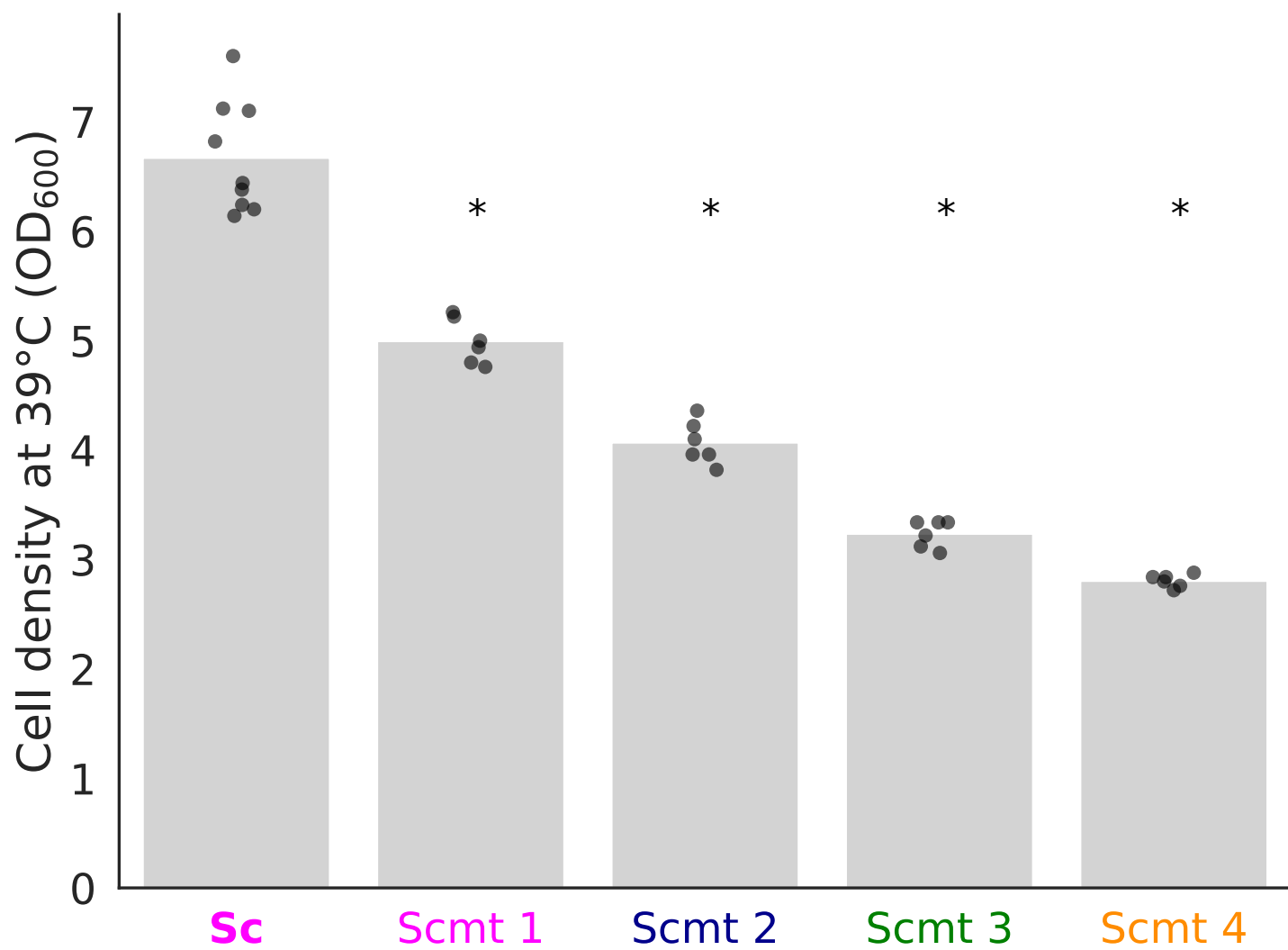

Supplement: iyaf233_Supplementary_Data [file iyaf233_supplementary_data.zip › Supplementary_Figure_7_GENETICS-2025-308167R1.pdf]

Sc

Sp

Sp+Scmt1

Sp+Scmt2

Sp+Scmt3

Sp+Scmt4

28°C

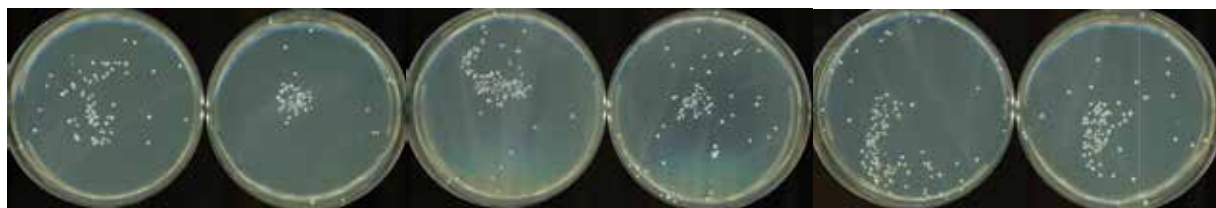

39°C

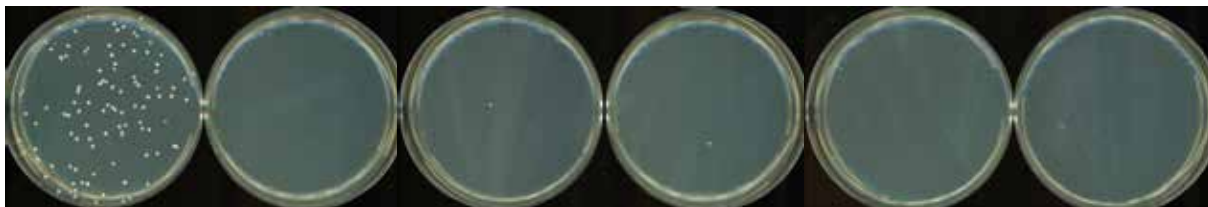

Supplement: iyaf233_Supplementary_Data [file iyaf233_supplementary_data.zip › Supplementary_Figure_8_GENETICS-2025-308167R1.pdf]

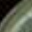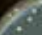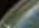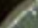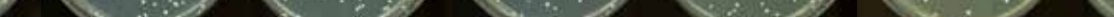

Supplement: iyaf233_Supplementary_Data [file iyaf233_supplementary_data.zip › Supplementary_Figure_9_GENETICS-2025-308167R1.pdf]
